# Traumatic Axonal Injury: Mechanisms and Translational Opportunities

**DOI:** 10.1016/j.tins.2016.03.002

**Published:** 2016-05

**Authors:** Ciaran S. Hill, Michael P. Coleman, David K. Menon

**Affiliations:** 1 John van Geest Centre for Brain Repair, University of Cambridge, Cambridge, UK; 2 Division of Anaesthesia, Department of Medicine, University of Cambridge, Cambridge, UK; 3 Wolfson Brain Imaging Centre, Department of Clinical Neurosciences, University of Cambridge, Cambridge, UK

**Keywords:** traumatic, axon, injury, brain, degeneration, therapeutics

## Abstract

Traumatic axonal injury (TAI) is an important pathoanatomical subgroup of traumatic brain injury (TBI) and a major driver of mortality and functional impairment. Experimental models have provided insights into the effects of mechanical deformation on the neuronal cytoskeleton and the subsequent processes that drive axonal injury. There is also increasing recognition that axonal or white matter loss may progress for years post-injury and represent one mechanistic framework for progressive neurodegeneration after TBI. Previous trials of novel therapies have failed to make an impact on clinical outcome, in both TBI in general and TAI in particular. Recent advances in understanding the cellular and molecular mechanisms of injury have the potential to translate into novel therapeutic targets.

## Trends

Multiple therapeutic targets are emerging that offer the potential to reduce secondary brain injury at a cellular level. These include cytoskeletal and membrane stabilisation, control of calcium flux and calpain activation, optimisation of cellular energetics, and modulation of the inflammatory response.

Wallerian degeneration, as occurs following an axonal injury, is an active, cell-autonomous death pathway that involves failure of axonal transport to deliver key enzymes involved in NAD biosynthesis.

Chronic microglial activation occurs following traumatic brain injury (TBI) and may persist for decades afterwards. This ongoing response has been linked to long-term neurodegeneration, particularly of white matter tracts.

Phagoptosis is the process whereby physiologically stressed but otherwise viable neurons are phagocytosed by microglia in response to a range of eat-me signals induced by tissue injury.

## TAI is a Common and Severe Subtype of TBI

**Figure 1:**
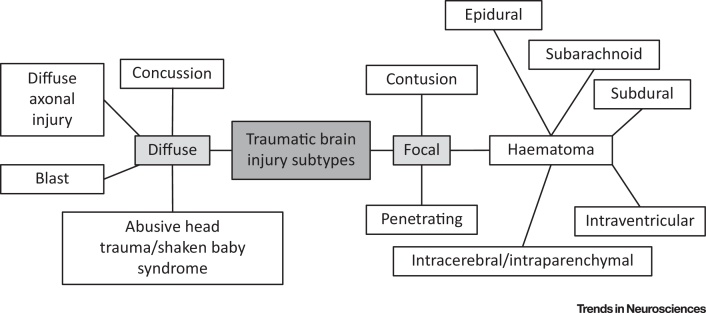
Traumatic Brain Injury Subtypes. There are several different variants of traumatic brain injury, which often coexist and have significant overlap. They can be broadly divided into focal and diffuse injuries, although it is worth noting that true focal injuries are rare and blast injuries lack a pure neuropathological correlate. The clinical presentation and prognosis of a traumatic brain injury varies depending on the individual nature of the injury. The inherent variability makes it challenging to establish the optimal treatment and there is recognition of the value of an individualised approach.

**Figure 2:**
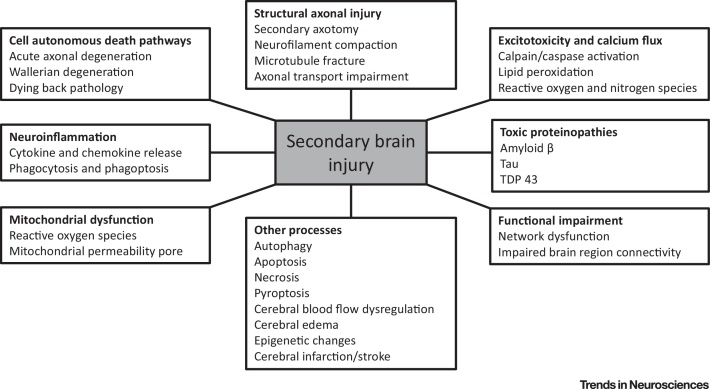
Cellular and Molecular Activities Resulting in Secondary Brain Injury. Following a traumatic insult to the brain, an extensive series of various cellular processes is initiated that leads to further neuronal dysfunction and death. This contributes to the complexity of traumatic brain injury but also provides a variety of therapeutic targets.

**TBI** (see Glossary) is a major public health concern that contributes to one-third of all injury-related deaths [1] (http://www.cdc.gov/traumaticbraininjury/data). TBI is an emerging research priority, with large North American and European comparative effectiveness research studies enrolling several thousands of patients and looking at a broad range of research questions 2, 3, 4. The definition of TBI is an ‘alteration in brain function, or other evidence of brain pathology, caused by an external force’ [5]. However, this unitary epidemiological definition encompasses a complex disease process with diverse injury subtypes that may overlap (Figure 1). There is an increasing drive to differentiate these subtypes to allow precision-medicine approaches to management, where specific pathobiological processes can be matched to mechanistically appropriate therapy. Such approaches also need to take account of differences in host response that arise from coexistent trauma reactions and pre-existing comorbidities. The temporal evolution of **secondary brain injury** and associated pathophysiological responses (Figure 2) that follow the **primary brain injury** are also important when trying to understand a TBI. This review examines key molecular mechanisms and potential therapeutic targets in one of the most common and severe types of TBI, traumatic or **diffuse axonal injury** [6]. The terminology regarding axonal injury is in flux but historically the cellular and animal models of this injury type have been referred to as **TAI** [6]. This review uses the term TAI to refer to studies of TBIs where axonal injury is the dominant component, regardless of whether they were undertaken in human or animals.

## Axonal Structure and the Initial Mechanical Injury

Axons can be up to ten thousand times the volume of the parent neuronal cell body and their elongated structure places them at particular risk of mechanical injury. An axon contains longitudinal tracks of microtubules arranged in a series of overlapping and highly dynamic strands that span the length of the neuron. Microtubules may offer some structural support but primarily act as polarised tracks for motor proteins. Neurofilaments provide tensile strength and their radial charges are thought to influence axonal diameter, while actin filaments provide additional membrane stability in the form of a regular repeating ring-like structure that runs around the circumference of the axon and is held together with spectrin links and adductin caps [7].

TAI results from high-velocity translational or rotational forces acting on the large, gyrencephalic human brain, typically due to a motor vehicle accident or fall. Inertial forces shear and stretch axons to breaking point/primary **axotomy** or partially damage them, triggering molecular pathways that result in secondary axotomy/axon degeneration [8]. It is generally accepted that primary axotomy may be an uncommon component of TAI and would most obviously be found with a tissue laceration or other direct injury [9]. An elongating stretch of at least 10% that occurs in 100 ms or less appears to represent a threshold for sublethal axonal injury with secondary consequences [10]. Some white matter bundles are more vulnerable due to their orientation and location (for example, the corpus callosum and brainstem) or at interfaces between tissue compartments of different density such as the grey–white matter junction [11]. Other modifiers of TAI susceptibility include local cellular features, including the stiffness of adjacent tissue, maximum diversion angle, and internal neuronal cytostructure [12]. Myelination may afford some degree of protection against TAI, through mechanisms that include metabolic support by glia, the greater physical robustness of myelinated axons and hence tolerance of greater injury forces, and better functional recovery 13, 14. An additional driver of variable resilience may be differences in the site of post-injury ionic fluxes. In myelinated axons the altered ionic gradients favour nodal and paranodal areas, whereas unmyelinated axons experience more uniform and widespread injury-induced ionic fluctuations [15].

The classical histological finding in TAI is of ‘retraction bulbs’, which are thought to develop following primary axotomy. The term may represent a misnomer, as they are now generally thought to represent the visualisation of an abnormally accumulated substance, such as the transmembrane glycoprotein amyloid β precursor protein (APP), due to impaired **axonal transport** rather than actual axonal retraction [16]. Primary axotomy is generally considered relatively rare in human TAI [6], but direct evidence quantifying this is limited. A second major type of morphological change typically seen in TAI is swelling or varicosities. These are regions of isolated or multiple axonal swellings (‘beading’) found along the length of an otherwise intact axon. These have been linked to microtubular dysfunction and fracture [16]. Microtubules have a viscoelastic nature and are particularly susceptible to breakage because, when rapidly stretched, they become the stiffest portion of the axon. These breaks lead to microtubular undulations and impairment of axonal transport with subsequent accumulation of axonal transport cargos such as βAPP 16, 17. Although similar undulations have occasionally been demonstrated in sham controls, the consensus is that these appearances provide good evidence of stretch injury 16, 18. However, not all instances of TAI show βAPP accumulation, and the true pathophysiological significance of axonal swellings is unclear, as is their reliability as a modifiable marker of effective neuroprotection [19]. Hanell *et al.* provide a review of the different axonal phenotypes seen in TAI and their potential significance [18].

Immunohistochemical analysis for APP accumulation is currently the gold-standard clinical and experimental technique for assessment of TAI [15]. However, in most instances this is a clinical/radiological diagnosis and does not require brain tissue. This diagnosis is based on a typical clinical history of a high-energy TBI coupled with conventional neuroimaging findings (with X-ray CT) that show no significant focal lesions [15]. MRI has shown marked improvements in diagnostic sensitivity for TAI in living patients and newer MRI approaches can identify either biomarkers of the axonal injury itself with diffusion tensor imaging or the microhaemorrhages that result from injury to the microvessels accompanying white matter using sequences such as gradient echo and susceptibility-weighted imaging 20, 21.

## Cytoskeletal Protection

Neurofilaments are the dominant intermediate filament of axons; they are produced in the neuronal soma and transported throughout the neurite. Structurally they are obligate heteropolymers assembled from a central rod domain surrounded by the neurofilament triplet proteins (which may be light, medium, or heavy) 22, 23. Neurofilaments may be a key contributor of axon tensile strength and resilience to mechanical stretch. However, it remains unclear whether they have additional roles beyond acting as a simple structural protein. Following injury, the axonal swellings found in TAI develop neurofilament accumulations of all subtypes [24]. Neurofilaments undergo a process of compaction whereby the interfilament spacing is reduced due to side-arm phosphorylation or proteolysis and increased density is found within axonal swellings [25]. This finding could be due to impaired axonal transport leading to accumulation of cargos, including neurofilaments. However, the failure of the neurofilament central rod-domain marker RM014, which is exposed during compaction, to co-accumulate with APP suggests that the compaction is more complex than a simple transport impairment 23, 26, 27.

The calcineurin inhibitor FK506 (tacrolimus) is used in humans as an immunosuppressive agent to reduce the risk of organ rejection. It inhibits phosphatases and hence attenuates the effects of dephosphorylation-dependent proteases on the neuronal cytoskeleton, including, neurofilament compaction and spectrin/ankyrin degradation 23, 28. The subsequent reduction in structural axonal injury and secondary degeneration in experimental models of TAI implies that this pathway is important in cytoskeletal breakdown, although the effects differ between axonal subpopulations and are particularly pronounced in unmyelinated axons 28, 29. A single dose of FK506 in a rat model of lateral fluid-percussion injury has been shown to reduce loss of dendritic spines and also axonal damage as measured with antibody labelling of βAPP 29, 30. Pretreatment of cultured primary cortical neuronal axons with FK506 1 h before an *in vitro* stretch reduced secondary axotomy [31]. FK506 has also been suggested as a treatment for post-traumatic epilepsy, which may result from increased calcineurin activity in the hippocampus [32]. FK506 in combination with hypothermia seems to protect axons in excess of isolated treatment in a rat lateral-percussion model [33]. Despite the current clinical use of FK506 as an immunosuppressive agent in humans there are still no results available for its effects in human TAI.

Microtubule stabilisers including paclitaxel (Taxol) have also been suggested as potential neuroprotective agents and there is evidence that they may affect the rate of axonal degeneration 17, 34, 35. Unfortunately, paclitaxel has poor blood–brain barrier permeability and serious side effects in humans, including peripheral neuropathy. Despite this there remains interest in this therapeutic avenue and Taxol-like agents (taxanes) may as yet prove to be of use [36]. Another agent thought to act on cytoskeletal proteins is epothilone D. This microtubule-stabilising drug is brain penetrant and shows evidence of modulating injury-induced axonal sprouting in cortical neuron cultures following experimentally induced traumatic axotomy [37]. However, despite emerging evidence for efficacy in spinal cord injury, data directly supporting efficacy in brain injury remains lacking 37, 38.

Spectrin is a key cytoskeletal element whose breakdown leads to the formation of specific, quantifiable, stable αII spectrin fragments of 145 kDa and 150 kDa (SBP145 and SBP150, respectively). The SBP145 breakdown product is brain specific and is found in contusions with brain necrosis, but an isolated TAI is also sufficient to stimulate its generation. The rise in breakdown products may occur within 15 min and is reliably demonstrated within 3–24 h 39, 40. SBPs have been proposed as a potential biomarker of brain injury (Box 1). There are no examples of direct spectrin stabilisers; prevention of spectrin breakdown by calpain inhibitors is well documented but the effects of reduced spectrin degeneration on axon survival in TAI remain largely speculative 41, 42.

Ankyrins are a family of adaptor proteins that link the spectrin–actin complex to integral membrane proteins, a function vital to the maintenance of ion channels within the plasma membrane. Proteolytic degradation of ankyrin-G following axonal injury may result in mislocalisation of sodium channels in nodal regions. It might also encourage instability of the axolemma through altered binding of neurofascin, a member of the L1 immunoglobulin superfamily of cell adhesion molecules 43, 44. Hence, direct stabilisation of ankyrin or reduction of its proteolysis may offer a new therapeutic avenue, if such an intervention could be safely achieved in humans.

## Cell-Autonomous Axonal Death Pathways

**Figure 3:**
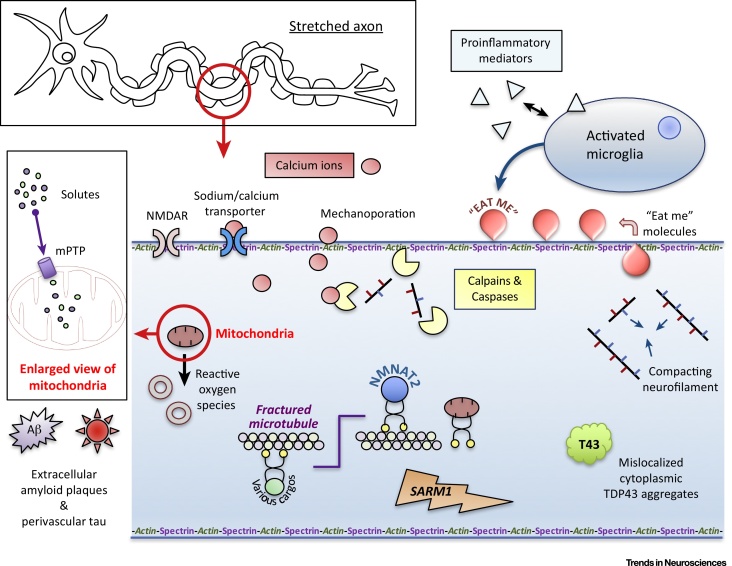
Key Figure: Summary of Molecular Mechanisms and Therapeutic Targets in Traumatic Axonal Injury (TAI)

Injury to axons in the central nervous system can lead to death of the whole neuron, although this may vary by neuronal subtype and with the distance of the injury from the cell body 45, 46. Loss of the distal axon will also hamper connectivity and the associated reduction in synaptic activity may influence overall neuron function even if plastic reorganisation ensues [47]. There are numerous pathways and pathophysiological processes involved in axon degeneration and neuronal death (Figure 3, Key Figure). These pathways differ by injury type and also with time from the injury but may eventually converge. Each process provides the opportunity for therapeutic intervention, ideally at a point before irreversible structural changes occur.

One mechanism proposed for the loss of axons when axonal transport is impaired is Wallerian-like degeneration (WLD). This is related to **Wallerian degeneration (WD)** (Box 2). WLD shows similarities to WD in molecular regulation and similarly involves granular disintegration of the axon segment distal to the injury site. Some neuronal cell types, such as the retinal ganglion cell, also suffer a proximal ‘dying-back’ pathology with soma injury or death after the initial insult 45, 48. Optic nerve stretch modelling has shown that some nerve fibres may also degenerate weeks to months after the injury. Whether this is a delayed form of WD (for example, following the death or significant impairment of the corresponding soma) or should be considered a separate process is still being elucidated 49, 50.

When subjected to a TBI, WLD^s^ mice (which show delayed Wallerian degeneration) demonstrate reduced physical evidence of TAI, less evidence of axonal transport disruption (swelling, APP accumulation, microtubule disruption), and delayed motor and cognitive impairment. These findings are consistent with Wld^S^-sensitive degeneration following TAI but this has not been definitively confirmed [51]. A recent study of closed head injury (weight drop) in SARM1 knockout mice has showed evidence of *in vivo* protection through inhibition of the WLD pathway, with reduction of behavioural deficits and axonal APP aggregates in the corpus callosum [52]. However, the authors were unable to quantify effects on axonal loss other than indirectly through phosphorylated neurofilament heavy chain levels [52].

There are no currently available modulators of SARM1. However, as the WD pathway is increasingly understood other therapeutic targets may emerge within the pathway and offer new treatment opportunities. One example is P7C3 and related compounds; this aminopropyl carbaxole discovered using an unbiased *in vivo* screening approach for neurodegenerative disease modifiers seems to be proneurogenic and antiapoptotic [53]. P7C3 is thought to bind to nicotinamide phosphoribosyltransferase (Nampt), possibly enhancing its activity [54]. Nampt is a rate-limiting enzyme important in WD that converts nicotinamide to NMN and subsequently NAD. Further *in vivo* work in models of blast injury and TBI have shown that P7C3 can provide neuroprotection and/or preservation of function 55, 56. However, there are still questions about the exact mechanism of P7C3 action and it remains unclear how the apparent neuroprotection provided by Nampt activation can be reconciled with the finding that the Nampt inhibitor FK866 has also been shown to delay WD [57].

## Mechanotransduction and Calcium Permeability

When a central nervous system axon is stretched there is an acute increase in intracellular calcium primarily derived from intracellular stores. This is followed by a more gradual and long-lasting dysregulation of intracellular calcium metabolism 31, 58. Although as yet unproved, it has been suggested that physical forces may lead to ‘mechanoporation’, a term variably used to refer to either direct or secondary opening of the axolemma, leading to intracellular fluxes of calcium 58, 59, 60, 61. Calcium has established roles in many forms of cell death, including the apoptotic death pathway and WD, and is therefore a potential therapeutic target in TAI. Increases in intracellular cytoplasmic calcium, such as might occur with an inflammatory response to a traumatic injury, disruption of energy metabolism, or damage to the cell membrane, can trigger a pathogenic cascade culminating in cell death [31]. A focal increase in calcium concentration in an axon precedes, and can result in, the development of axonal spheroids [62]. Conversely, spheroid formation can be prevented by the blockage of NCX1, N-, or L-type voltage-gated calcium channels, possibly by preventing a threshold level of axolemmal calcium from being reached [62]. While the benefit of preventing such ultrastructural changes remains unproved, it is evident that an unchecked increase in intracellular calcium levels can trigger secondary axotomy. The mechanism by which this is likely to occur includes active destruction of the cytoskeleton mediated by calcium-dependent calpains, caspases, cysteine proteases, and phosphatases in response to cytosolic accumulation of calcium [63]. Additionally, mitochondrial sequestration of calcium can result in energetic dysfunction, generation of reactive oxygen species, and subsequent oxidative damage [23].

Attempts to use agents such as Kollidon VA64 to reseal microdefects in the plasma membrane following injury aim to guard against mechanoporation-related calcium damage. Initial *in vivo* results with this agent in rodent models of TBI have been encouraging [64]. Alternative membrane-resealing agents include poloxamer 188. This non-ionic surfactant has been shown to inhibit apoptosis and necrosis *in vitro* after a stretch injury and exhibits neuroprotective effects following TBI in animal models but has not been tested in humans. The exact mechanisms of action of this agent are debated but may partially involve inhibition of p38 mitogen-activated protein kinase (p38-MAPK) activation or cathepsin B- and tBid-mediated mitochondrial cell death triggering [65]. Other ‘membrane-sealing’ compounds are currently undergoing preclinical testing, including PEG-PDLLA micelle treatment, polyethylene glycol, and tripartite motif (TRIM) proteins like Mitsugumin 53 66, 67, 68. A barrier to clinical translation in this context is the rapidity of calcium rise following injury, which makes direct prevention of early calcium entry problematic.

## Calpains as a Convergence Point in Axonal Degeneration

Excitotoxicity has a long history of being implicated as a secondary brain injury mechanism contributing to neuronal death following trauma. Excitotoxic cell death is intimately linked to downstream calcium ion influx modulation and dysregulation, which is mediated in part by calpains [69]. Extracellular glutamate levels have been shown to be elevated in both experimental and human TBI, but the failure of glutamate antagonists in clinical trials has resulted in interest shifting to downstream targets, including calpains [70]. Calpains are calcium-dependent, non-lysosomal cysteine proteases. Their baseline activity is low and in normal physiological conditions they are predominantly involved in cell signalling and plasticity [42]. In response to axonal injury and associated calcium shifts, calpains move from the cytosol to the plasma membrane, where sustained activation causes widespread proteolysis. Several membrane, adhesion, and structural cytoskeletal proteins are targeted by calpains, including the key cytoskeletal protein spectrin. Calpains have been strongly implicated in the later stages of WD in both the peripheral and central nervous system [69]. Their ability to degranulate the distal segment of injured axons may be partly due to *in vivo* proteolysis of neurofilaments, although the exact steps in such a mechanism have not been fully described [71]. Calpain activity within motor nerve terminals at the neuromuscular presynaptic junction may also cause denervation, although whether this precedes or follows axonal degeneration is unclear [71]. While calpain inhibition has shown robust morphological protection this has not extended to electrophysiological function *ex vivo* – a failing that may limit human translation [71]. Continued interest in this pathway is driven by the belief that widespread calpain activation may be an early mediator of additional secondary brain injury in TAI and by the fact that calpain-induced spectrin breakdown provides specific molecular biomarkers of the process [72] (Box 1). Subaxolemmal calcium-induced calpain-mediated proteolysis may contribute to the axolemmal permeability observed in TAI. Calpains may also cause long-term deficiencies in axonal transport and plasticity leading to persistent dysfunction and poor clinical recovery. Recognition of these potential roles in ongoing degeneration has focussed efforts on inhibition of calpain as a therapeutic strategy, primarily aimed at affording cytoskeletal protection. This may be achieved either directly with calpain/protease inhibitors or indirectly by reducing intracellular free calcium [42]. Numerous compounds that target excitotoxic calcium-, calpain-, or caspase-related mechanisms have been investigated in the context of TAI and TBI. A range of these has progressed to human trials, many based around targeting glutaminergic excitotoxicity mediated by the NMDA receptor. Examples include magnesium sulfate [73], selfotel (CGS 19755) [74], dexanbinol [75], and amantadine [76]. Unfortunately, none of these interventions have shown benefit in clinical TBI. Several calpain- and caspase-based agents have been trialled in rodent models with mixed success but these have not progressed to human trials. Although there remains hope that some of these therapies may find use in clinical practice, they share common inherent limitations, including unwanted modulation of otherwise beneficial cell activities, and logistic difficulties around the need for early drug administration.

## Mitochondria and Energetics in TAI

Axonal stress is associated with reduced mitochondrial movement, disruption of cristae, and swelling, coalescence, or fragmentation of mitochondria [77]. Axons are sensitive to energy depletion and utilise ATP to sustain membrane potentials and ionic gradients, prevent abnormal calcium influx, and sustain transport of cargos (including mitochondria) within the axon. After primary axotomy, mitochondrial respiration and glycolysis fall 78, 79, resulting in a decline in ATP levels that can contribute to irreversible axonal damage 79, 80, although a recent report indicates that loss of mitochondrial membrane potential can be quite a late event [81]. Modulation of energetic failure remains a potential therapeutic target in TAI, either directly to ameliorate axonal energy failure or to address mitochondria-related injury. There are several ways in which mitochondrial dysfunction may contribute to axonal pathology. When subjected to injury, axons are sites of reactive oxygen species production, energy failure, and mitochondrial permeability pore (mPTP) generation [82]. The mPTP is an inner membrane protein that is induced in response to increased calcium matrix concentration and allows movement of small molecules (<1.5 kDa) in or out of the mitochondria, a process that may lead to swelling and death 83, 84. The view that the mPTP participates in axonal degeneration has led to its study as a potential therapeutic target in TAI. Cyclosporin A is a commonly used immunotherapeutic drug that binds and inhibits cyclophilin D, a protein complex purported to be involved in the opening of the mPTP [85]. Cyclophilin D knockout mice show decreased numbers of distal, but not proximal, axonal bulbs and varicosities in a fluid-percussion mouse model of TAI [18]. Results with cyclosporin A administration in TAI models are mixed. Pretreatment with cyclosporin A failed to reduce axonal swelling in primary neuronal cultures exposed to reactive oxygen species [62]. However, cyclosporin A does attenuate cytoskeletal changes and axon degeneration after a mild axon stretch injury, while intrathecal administration reduced delayed axotomy in a rat acceleration model [86]. Human Phase II trials of cyclosporin are currently underway. A similar approach uses the cyclosporin analogue NIM811, which also binds to cyclophilin D and prevents the formation of the mPTP [87]. NIM811 reduces calpain-mediated spectrin degradation, neuronal degeneration, and cognitive deficits when administered up to 12 h after a traumatic injury 88, 89. FK506 (tacrolimus) may also have a role in modulating cellular energetics, since it inhibits calcineurin and hence reduces translocation of BAD to BCL-X in mitochondria, with reduction of mPTP opening [15].

Attempts to improve mitochondrial energetics have included the use of *N*-acetyl cysteine [90] which has shown potential symptomatic benefit when used in mild military blast injury [91]. Exposure to low-level laser light or 670-nm light provides a novel non-pharmacological approach to improve mitochondrial energetics through alteration of redox state and transcription factor expression while inducing modest increases in nitric oxide and reactive oxygen species. This technique has the potential for human applications and, in addition to isolated case studies, has shown benefit in animal models 92, 93. There may be scope to combine this technique with systemic administration of methylene blue, a compound that interacts with numerous targets, acts as a redox cycler, and upregulates mitochondrial energetics [94]. Methylene blue may also promote autophagy, reduce brain oedema, and inhibit microglial activation 95, 96.

Another mitochondrial target identified with *in vitro* stretch modelling is cardiolipin peroxidation. The compound XJB 5131 has been shown to reduce lipid oxidation and caspase activation in a rat cortical contusion model, with subsequent improvement in both lesion size and functional measures [97]. Further research is still required to characterise the compound before its use beyond rodent models can be considered.

Creatine is an endogenous amino acid, administration of which can increase stores of the high-energy metabolite phosphocreatine. Creatine may directly act on central nervous system axons and modify response to brain injury by inhibiting calcium-induced activation of the mPTP, maintaining ATP levels, preserving normal mitochondrial membrane potentials, and reducing intramitochondrial calcium levels [98]. A randomised pilot study in children with severe TBI was encouraging, with some prevention of post-traumatic symptoms and improvement in several parameters including length of intensive care unit stay and cognition [99].

## Inflammation and Microglial Phagocytosis and Phagoptosis

TAI triggers a complex cascade of inflammatory response with release of cytokines, chemokines and growth factors by microglia, astrocytes, and neurons [100]. Many cytokines and chemokines are capable of inducing mixed pro- and anti-inflammatory effects under specific conditions. This duality of function makes it difficult to disentangle their beneficial and detrimental effects [101]. Thus far, despite often promising preclinical results, agents that modulate the inflammatory response have failed to deliver clinical benefit in human TBI. Prominent examples include corticosteroids and anti-tumour necrosis factor (TNF) therapy 75, 102. Blocking of the ‘proinflammatory’ interleukin (IL)-1 receptor remains a therapeutic target and is the subject of ongoing investigation in the form of a Phase II clinical trial that is examining IL-1ra [103].

Purinergic signalling through cognate transmembrane receptors represents a core part of the neuroinflammatory response. Adenosine signals via P1 receptors while ADP and ATP activate P2 receptors. Stretched cells, including astrocytes, can release their intracellular stores of ATP, which activates ionotropic P2X_7_ receptors localised to astrocytic end feet and colocated with aquaporin 4 expression. P2X_7_ activation leads to IL-1β-mediated exacerbation of local neuroinflammation, reactive gliosis, and cytotoxic oedema. It also results in membrane poration and increased tissue damage as a result of enhanced calcium influx 104, 105. Brilliant Blue G (BBG) directly antagonises P2X_7_ and might downregulate this damaging response up to 4 h post-injury [106]. The compound is nontoxic and has shown promise in attenuating optic nerve crush injuries and rodent TBI models, although the effects have been mild and not all results have been reproducible 106, 107.

Following TBI there is rapid and widespread activation of microglia. Temporally, the numbers of microglia appear to follow a multiphasic pattern with early and late peaks [108]. Beyond the acute inflammatory phase, chronic microglial activation is triggered that can persist for months or years. This has been shown in murine models 1 year following experimental brain injury and in human subjects with the PET ligand [^11^C](R)PK11195 109, 110. Post-mortem examination of the brains of TBI survivors who die of other causes at varying intervals after injury demonstrates ongoing activation years and decades after the original insult, in association with regions of substantial white matter volume loss [49]. This has been proposed as a potential mechanism linking TBI and late neurodegeneration, including **Alzheimer's disease (AD)** (Box 3). Similarly to the cytokine/chemokine response, microglia can be polarised within a spectrum that includes classical/proinflammatory and alternative/reparative phenotypes with different mechanistic impacts 111, 112. Understanding this balance and the complexities of mixed phenotypes so that they can be modified in a beneficial way is an important challenge in TAI research.

Phagocytosis and debris clearance is required for maintenance of tissue homeostasis, regeneration, and plasticity but may be detrimental when occurring in excess [113]. Severing of neurites or traumatic injury causes translocation of phosphatidylserine residues from the inner plasma membrane to the cell exterior. This is one of several ‘eat-me’ signals in injured neurons, recognised by activated microglia, and can trigger phagocytosis of the injured neuron [114]. More recently, the concept of **phagoptosis** has emerged. Molecules that are expressed by stressed neurons and known to directly trigger the phagoptotic response include phosphatidylserine and desialylated glycoproteins. Alternatively, opsonins and receptors including MertK, MFG-E8, galectin-3, protein S, and GAS6 can act as intermediaries 114, 115. Counter-regulatory ‘don’t-eat-me’ signals also exist and include neuraminidase 114, 116. Although this description of microglia-mediated injury is plausible, we do not know yet whether such phagoptosis signals are displayed in response to TAI. If this injury mechanism is confirmed, modulation of phagoptosis could potentially allow rescue of these partially but not irreversibly injured neurons. Whether these cells could then recover and functionally reintegrate in a beneficial manner would be an important subsequent question.

## Concluding Remarks: Mechanistic Understanding May Translate to Therapies

Despite increasing interest in and research into TAI, there has so far been a notable lack of translation into efficacious patient therapies [70]. The extensive range of *in vitro* and *in vivo* models that exists to examine various aspects of TAI has contributed to substantial advances in mechanistic knowledge of axonal injury and death pathways [117]. However, because of inherent limitations of model systems it is critically important to explore and compare pathophysiology in human TBI, for example through **cerebral microdialysis** and imaging technologies, to be certain that results obtained in TBI models are applicable in clinical settings. Crucial questions remain but the hope is that increased knowledge gained from an improved mechanistic understanding of injuries will translate into effective therapies and improved clinical outcomes.

Outstanding Questions Is increasing total neuron survival the optimal target to improve outcomes in TAI or are poor outcomes more a failure of function and connectivity at the level of the individual cell or large network?

Why is there progressive white matter volume loss following TAI and does microglial phagoptosis contribute?

To what degree is primary or secondary axotomy responsible for dysfunction following TAI?

Do degenerating axons trigger death in adjacent axons? If so, how is this mediated?

How much does Wallerian-like degeneration contribute to axonal death in TAI and can WLD^s^ or SARM1 knockout offer protection in model systems (Box 2)?

Why do different axons degenerate at different rates?

Can stabilisation of the axonal structure protect the axon from injury or reduce the rate of secondary axotomy?

What is the functional significance of axonal varicosities and can they be repaired?

Does TAI lead to significant axonal transport impairment at varicosities and/or more widely and does this contribute to the development of neurodegenerative disease?

Are histological subtypes of axons seen in injury (e.g., APP positive and negative) of mechanistic or prognostic importance?

How does TBI contribute to the development of neurodegenerative diseases (Box 3)?

Why do proteins including amyloid β and TDP43 increase following TAI and are these detrimental?

Can aspects of the inflammatory (cytokine/chemokine) response be modulated to alter neuronal survival and patient outcomes?

What is the role of autophagy in TAI?

What is the optimal biomarker for TAI (Box 1)?

## Box 1 Molecular Biomarkers in TAI

An effective biomarker of axonal injury would provide a diagnostic tool, a quantitative measure of injury severity, and an indicator to gauge treatment efficacy. The ideal biomarker would be highly sensitive and specific for TAI, rapidly reflect changes in injury status, and be easily obtained, such as from a peripheral blood sample. Axon degeneration results in the generation of cytoskeletal breakdown products and several of these have been explored as potential biomarkers. Calpains and caspase-3 irreversibly cleave αII spectrin, a neuronal membrane-associated scaffolding protein, into fragments of various molecular weights. These αII spectrin breakdown products (SBDPs) are found in human cerebrospinal fluid (CSF) and blood following severe TBI and may predict injury severity and outcome. However, a rise in SBDPs is not specific for TAI and may also be found in cerebral ischaemia, neurodegenerative diseases including AD, and normal ageing 72, 118. Neurofilament light chains are another key cytoskeletal protein with some specificity for TAI [119]. Axotomy releases phosphorylated neurofilaments into the blood and CSF that may correlate with injury severity [120]. A third element to be explored as a biomarker is glial fibrillary acidic protein (GFAP) and its breakdown products. GFAP is a brain-specific intermediate filament that is prevalent in astrocytes and correlates with intracranial injury [121]. Other cytoskeletal products that are being investigated include the microtubule-stabilising protein tau and microtubule-associated protein 2 (MAP2) [119]. Many non-cytoskeletal molecules are also being investigated as potential biomarkers in TBI; these include S100-β (a 21-kDa calcium-binding protein), neuron-specific enolase (a 78-kDa γ-homodimer glycolytic enzyme isomer), and the neuron-specific protein ubiquitin C-terminal hydrolase (UCH-L1). An alternative approach to biomarker detection is to characterise functional responses to injury. Cerebral microdialysis in both experimental models and human TBI captures the generation of a wide range of pro- and anti-inflammatory cytokines and chemokines in response to brain injury. However, the complex pattern of production with temporal variation, covariance, and multiple interactions makes their interpretation and use as biomarkers challenging 101, 122. Despite active research, biomarkers that are specific for axonal injury, as opposed to nonspecific markers of brain injury, are lacking, and an ideal marker remains to be identified.

## Box 2 Molecular Control of WD

The full molecular mechanism of WD is still being determined. One evolving model suggests that a key step in WD is failure to deliver the enzyme nicotinamide mononucleotide (NMN) adenylyltransferase 2 (NMNAT2), an essential survival factor, from the neuronal soma to the distal axon. Loss of NMNAT2 beyond the site of injury due to either interrupted transport or transection of the axon leads to a reduction in the ubiquitous coenzyme NAD and an increase in its precursor NMN. Mice expressing a mutant slow WD protein (WLD^s^) that provides a more stable form of NMNAT activity in the axonal periphery are less dependent on ongoing transport of NMNAT2 from the neuronal soma and exhibit markedly slower Wallerian-like degeneration [123]. It has been proposed that it is NMN that may ultimately lead to the triggering of WD [57]. Other possibilities include a direct effect of NAD depletion or some combination of the two [124]. A crucial downstream regulator of this intrinsic axonal self-destruct pathway is the sterile alpha and TIR motif-containing 1 (SARM1) protein. SARM1 mediates rapid fragmentation of severed axons and when this factor is knocked out axons show a robust delayed degeneration phenotype that is comparable with WLD^s^ 80, 125. The mechanism by which SARM1 regulates axon degeneration is still being elucidated, but recent data indicate that SARM1 either acts downstream on the NMNAT pathway or regulates a separate branch that converges at a downstream point 124, 126.

## Box 3 Links between TAI and Neurodegeneration

TBI is a major environmental risk factor for the development of AD [127] and chronic traumatic encephalopathy (CTE) [128] and has been implicated in frontotemporal dementia and motor neuron disease (MND)/amyotrophic lateral sclerosis (ALS), albeit inconsistently 129, 130. There are various potentially significant mechanistic links between TAI and AD, mostly converging around amyloid β and tau generation. TAI is thought to directly disrupt microtubule dynamics creating an impairment of axonal transport that subsequently leads to the characteristic histological finding of accumulations of APP cargo [16]. APP can undergo cleavage into amyloid β peptides, a key feature of AD, that aggregate into amyloid plaques. Some of these amyloid β species are directly neurotoxic, with oligomers including Aβ42 being particularly implicated [131]. Cortical-impact modelling has shown that injury can induce aggregation and oligomerisation of amyloid in a triple-transgenic AD mouse model [132]. Casualties of severe TBI may have increased brain amyloid levels, as detected by PET or histology several years following injury, that could correlate with outcome 19, 133, 134. In contrast to the PET findings, reductions in CSF levels of Aβ42 have been noted following TBI. This decline may be due to a temporary reduction in neuronal activity due to dysfunction, leakage across an opened blood–brain barrier, or deposition into insoluble plaques [135]. Tau is a microtubule-associated protein that, along with amyloid β, is considered by many to be a primary component of AD pathology. In AD, tau is seen to form hyperphosphorylated aggregates called neurofibrillary tangles. Increased tau levels in the extracellular space following severe TAI correlate with reduced amyloid β levels and adverse outcomes [136]. Recently, acute neuronal production of *cis*-phosphorylated tau (cPT) was found following TBI in mice and in hypoxic/serum-starved neurons *in vitro* [137]. It has been proposed that cPT is pathogenic. Pretreatment or post-injury treatment of mice with a cPT antibody blocked the increase in cPT and restored a range of structural and functional sequelae including risk-taking behavior and brain atrophy 137, 138. This provides an example of a novel potential biomarker and therapy that has shared implications for TAI and sporadic neurodegenerative disease. CTE is an increasingly recognised neurodegenerative condition that occurs following repeated TBI, often in the context of contact sports or military blast. The pathological mechanism is still being elucidated but the neuropathological hallmarks of perivascular tau accumulation and mislocalised cytoplasmic aggregation of transactive response DNA-binding protein 43 kDa (TDP43) may suggest common links and therapeutic avenues with sporadic neurodegenerative diseases like frontotemporal lobar degeneration (FTLD) and MND/ALS [139].

## Glossary

Alzheimer's disease (AD): a common dementia syndrome that clinically manifests as cognitive impairment, especially memory impairment and behavioural change. Pathologically it is characterised by amyloid plaques and neurofibrillary tangles of tau.
Axonal transport: the cellular process that moves proteins, lipids, vesicles, organelles, and other cargos along the length of an axon. Many cargos are trafficked bidirectionally (both away from and towards the soma) by motor proteins that move along microtubules or other cytoskeletal structures found within the cytoplasm.
Axotomy: a complete physical break in an axon, often caused by a stretch injury or laceration. Primary axotomy occurs at the moment of trauma while secondary axotomy occurs later due to secondary injury cascades/axon degeneration.
Cerebral microdialysis: a technique for sampling the molecular components of brain extracellular fluid by using a dialysate solution to establish concentration gradients across a semipermeable membrane. Involves the insertion of a sampling microdialysis catheter into a region of non-eloquent brain parenchyma.
Diffuse axonal injury: a type of human TBI characterised by a diffuse distribution of predominantly secondary axonal injury. A rapid axonal stretch injury triggers secondary axonal changes that can vary in extent and severity. A recent clinical definition requires human brain imaging to show four or more separate foci of signal abnormality (http://www.commondataelements.ninds.nih.gov).
Phagoptosis: the process where physiologically stressed but otherwise viable neurons are phagocytosed by microglia in response to a range of eat-me signals induced by tissue injury.
Primary brain injury: the brain injury that occurs at the moment of impact. On a macroscopic level, this includes contusions, lacerations, and haemorrhages. On a cellular level, primary axotomy can be included in this classification.
Secondary brain injury: all deleterious aspects of the brain injury cascade that occurs following the immediate traumatic injury to the brain. Secondary injury includes processes that begin seconds after the injury and may still be evident decades later. These include bleeding, energy failure, excitotoxicity, calcium influx, WD, phagocytosis, and many others.
Traumatic axonal injury (TAI): the *in vivo* or *in vitro* model equivalent of diffuse axonal injury. Typically emulated through the application of a mechanical stretch insult. The term is also used to refer to human TBI cases where there are one to three separate foci of signal abnormality (http://www.commondataelements.ninds.nih.gov).
Traumatic brain injury (TBI): an alteration in brain function or other evidence of brain pathology caused by an external force. This includes several other subtypes of TBI including focal and diffuse areas and patterns of injury.
Wallerian degeneration: a carefully controlled, active, cell-autonomous, and evolutionarily conserved death pathway that is distinct from other death mechanisms including apoptosis and necrosis. It involves the rapid granular fragmentation of an axon distal to the injury site following transection, typically occurring after a latent period of 24–48 h when modelled *in vivo* or 4–8 h *in vitro* (Box 2).
